# The Synergistic Effect of Vitamin C Supplementation and Early Feed Withdrawal on Heat Stress Mitigation in Broiler Chickens

**DOI:** 10.3390/ani15202996

**Published:** 2025-10-16

**Authors:** Hanan Al-Khalaifah, Nancy N. Kamel, Sherin Gabr, Ahmed Gouda

**Affiliations:** 1 Environment and Life Sciences Research Center, Kuwait Institute for Scientific Research (KISR), P.O. Box 24885, Kuwait City 13109, Kuwait; 2 Department of Animal Production, National Research Centre, El Buhouth St., Giza 12622, Egypt; black_tiger2167@yahoo.com; 3 Department of Poultry Breeding Research, Animal Production Research Institute, Ministry of Agriculture, Giza 12611, Egypt; sherygabr7@gmail.com

**Keywords:** Vitamin C, feed withdrawal, production performance, metabolic markers, antioxidant enzymes, gene expression, economic efficiency

## Abstract

**Simple Summary:**

Fast-growing broilers are highly susceptible to heat stress, which significantly disrupts their productivity, physiological functions, and overall profitability. Various mitigation approaches have been explored to counteract heat stress in broilers, with nutritional interventions being particularly attractive due to their practicality and cost-effectiveness. Among these, feed withdrawal and vitamin C supplementation are well-established strategies that have demonstrated efficacy under heat stress conditions. However, their combined effects remain insufficiently studied. Consequently, we aimed to evaluate the individual and combined properties of these two strategies. The results revealed that integrating feed withdrawal with vitamin C supplementation yielded superior outcomes, including higher weight gain, improved feed efficiency, upregulation of hepatic antioxidant enzyme expression, and better economic efficiency. These findings suggest that combining multiple stress mitigation strategies can enhance broiler productivity and profitability by supporting physiological homeostasis under heat stress conditions.

**Abstract:**

Mitigating stress responses is crucial for maintaining optimal productivity and profitability in modern poultry production. The effects of early feed withdrawal (EFW) and vitamin C (Vit C) supplementation, both individually and in combination, on heat-stressed broilers’ productivity, stress responses, and metabolic markers were investigated. In total, 240 newly hatched Cobb-500 male chicks were randomly distributed to four treatment groups (six replicates × 10 birds per group). A basal diet was offered to the control group; meanwhile, the EFW group underwent a 24 h early feed withdrawal on day four of age. The Vit C group was given 200 mg/kg vitamin C daily, and the EFW + Vit C group received both interventions. The study was executed under hot summer conditions, where the average minimum and maximum temperature-humidity Index (THI) were 29.15 ± 0.78 and 33.34 ± 0.76, respectively. The results demonstrated a superior stress-mitigating effect when EFW was combined with Vit C supplementation, leading to a significant improvement in productive parameters and elevated blood metabolic hormone levels. Additionally, total antioxidant capacity was enhanced, hepatic endogenous antioxidant enzyme expression was upregulated, and stress biomarkers were reduced. Furthermore, the economic efficiency indicators were significantly improved with EFW, and when EFW was combined with Vit C addition. These findings suggest that integrating multiple stress mitigation strategies, such as EFW and Vit C supplementation, may be more effective in relieving the adverse effects of heat stress by restoring homeostasis and optimizing broilers’ productivity and profitability.

## 1. Introduction

After decades of genetic selection for rapid growth, broiler chickens have become increasingly susceptible to heat stress (HS) owing to their higher production and metabolic rate, which can significantly impact their general health and productivity [1,2,3]. Fast-growing broilers are more vulnerable to HS and associated welfare challenges [4,5]. Recently, HS imposed a serious threat to the poultry industry, negatively impacting key physiological functions and overall profitability. Elevated environmental temperatures beyond the thermoneutral zone disrupt homeostasis, reduce feed intake, impair nutrient absorption, and induce both immunosuppression and oxidative stress [6,7,8]. This physiological imbalance directly compromises growth rates, feed efficiency, and carcass quality, causing substantial economic losses.

To mitigate the undesirable impacts of HS, it is crucial to implement effective management or nutritional strategies. One promising approach is the inclusion of potent antioxidant supplements, such as vitamins and probiotics, to alleviate oxidative stress and enhance broiler resilience [9,10,11,12,13,14]. Vitamin C, a potent antioxidant vitamin with metabolic bioactivity, plays a vital part in enhancing immune function, reducing oxidative damage, and improving the overall performance in heat-stressed broilers [1,15,16]. As a potent antioxidant, vitamin C scavenges free radicals, stabilizes cellular integrity, and alleviates oxidative stress induced by elevated temperatures [17,18]. Additionally, it supports energy metabolism, immune function, and stress resistance, reducing mortality rates and enhancing welfare during high-temperature conditions [8,19,20]. Although poultry can endogenously synthesize vitamin C, its production becomes insufficient under HS due to increased metabolic demands and excessive reactive oxygen species (ROS) formation [21]. Dietary vitamin C addition, at appropriate levels, has been shown to enhance broiler performance by enhancing feed intake, nutrient digestibility, antioxidant capacity, and immune response while mitigating heat-induced physiological disruptions [21,22]. Several studies have reported enhanced growth performance and elevated stress resistance biomarkers in broilers supplemented with vitamin C, particularly under heat stress conditions [3,15,23].

Other mitigation approaches have been proposed, including various early physiological adaptation strategies. Thermal manipulation [24,25] and feed withdrawal [26] during early life have shown promising evidence in reducing the impact of HS in later growth stages. Our previous research demonstrated that early feed withdrawal (EFW) for only 24 h promotes production performance in heat-stressed broilers [26]. Additionally, feed withdrawal during early development has been shown to enhance broiler chickens’ adaptability to HS by improving thermotolerance and reducing oxidative stress [26]. When multiple stress mitigation strategies are combined, a synergistic effect has been observed [7,25,27,28]. The consistency of these synergistic interactions across different nutritional strategies remains uncertain; however, combining them may provide a more comprehensive approach to managing HS in broiler chickens.

Combining different stress mitigation approaches in parallel may be more effective in alleviating stress [29]. The EFW and vitamin C supplementation in combination can offer a synergistic approach to managing HS. EFW modulates key physiological processes [26], while vitamin C enhances cellular defense against oxidative damage [30]. Together, these strategies can improve broiler performance, welfare, and profitability. Thus, this investigation introduces a novel synergistic strategy that combines early feed withdrawal and dietary vitamin C supplementation to enhance broiler resilience, productivity, and economic efficiency under heat stress conditions. The study aimed to evaluate the individual and combined effects of a 24 h early feed withdrawal and vitamin C supplementation in broiler reared under hot summer conditions. Specifically, we evaluated their impacts on production performance, physiological adaptation, stress biomarkers, and hepatic antioxidant marker gene expression, thereby contributing to the advancement of effective heat stress mitigation strategies.

## 2. Materials and Methods

### 2.1. Ethical Considerations

All the underwent trials were approved by the Institutional Animal Care and Use Committee, Animal Research Institute, Agricultural Research Center, Ministry of Agriculture and Land Reclamation, Egypt (Approval No. ARC-NRC-56-25).

### 2.2. Experimental Conditions

In total, 240 one-day-old Cobb-500 male chicks were randomly assigned to four investigational groups, each containing six replicates. The investigational groups were as follows: Control group, fed the basal diet (control); early feed withdrawal group (EFW), fed the basal diet with birds subjected to 24 h feed withdrawal on the 5th day of age; vitamin C group (Vit C), fed the basal diet supplemented with 200 mg of vitamin C/kg diet; early feed withdrawal and vitamin C supplemented group (EFW + Vit C), fed the basal diet, subjected to early feed withdrawal, and supplemented with vitamin C at 200 mg/kg. The feed withdrawal protocol (24 h on day 5 of age) was applied according to our previous findings [26]. Vitamin C was supplemented in the form of L-ascorbic acid phosphate powder (ROVIMIX^®^ STAY-C^®^ 35, DSM Nutritional Products Inc., Parsippany, NJ, USA). Feed was provided *ad libitum*, with unrestricted access to fresh and clean water at all times, except for EFW-exposed groups, which were subjected to 24 h feed withdrawal on day five of age. The basal diet, based on corn and soybean meal, was formulated according to the recommendations of NRC [31] and the Cobb-500 broiler management guide. The starter diet was offered from day 1 to 21, containing 2900 kcal/kg metabolizable energy (ME) and 23% crude protein (CP). From day 22 to 35, a grower-finisher diet was offered, containing 2951 kcal/kg ME and 21% CP. The exact ingredients and chemical composition of the basal diets were previously reported [26]. Chicks were raised in a tiered cage system, partitioned with wire mesh into uniform compartments (1.0 × 0.50 × 0.40 m), each accommodating ten birds. Lighting was provided for 23 h per day during the first three days of chicks’ life, and then reduced to 16 continuous hours per day for the remainder of the experiment.

**Figure 1:**
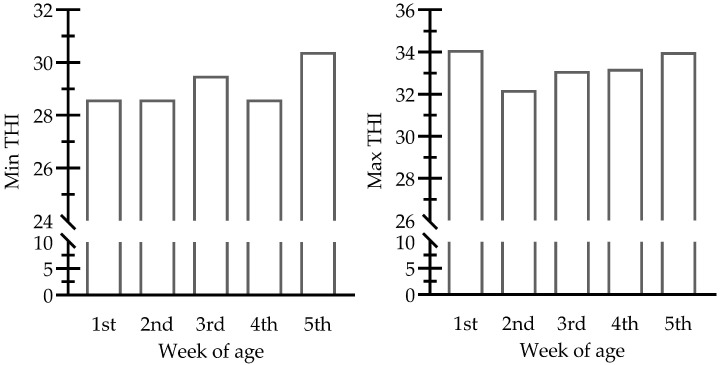
Weekly changes in temperature-humidity index (THI). Min: minimum, Max: maximum.

The experiment was carried out under hot summer conditions. Ambient Relative humidity and maximum/minimum daily temperatures were recorded. The temperature-humidity index (THI) was determined using the formula of Marai, et al. [32] (Figure 1). THI was interpreted according to the classification of stress levels based on Dedousi, et al. [33] (i.e., 27.8 to <28.9 moderated, 28.9 to <30.0 severe, and ≥30 very severe stress).

### 2.3. Production Performance

The body weight (BW) of the birds was documented on day 1 (initial BW) and day 35 (final BW) of the experiment to determine body weight gain (BWG). Meanwhile, the feed intake (FI) was recorded per group replicate, and the feed conversion ratio (FCR) was calculated accordingly.

### 2.4. Blood Sampling and Analysis

At the end of the experiment, blood samples were collected from the wing vein (n = 6; one bird per group replicate). Blood hemoglobin (Hb) was measured immediately according to Jain [34]. Serum was collected by centrifugation of blood samples at 4000 rpm for 15 min and was kept at −20 °C until further analysis.

The thyroid hormones (triiodothyronine, T_3_, and thyroxine, T_4_) were measured in serum using commercial radioimmunoassay (RIA) kits (Byk-Sangtec Diagnostica, Dietzenbach, Germany, Immulite 2000, DPC, Los Angeles, CA, USA) [35]. Meanwhile, serum total protein (TP) and albumin (Alb) levels were estimated according to Weichselbaum [36] and Doumas, et al. [37], respectively. Globulin level was computed as the difference between TP and Alb.

Serum lipid profile was assessed using commercial colorimetric kits (Egyptian Company for Biotechnology, Cairo, Egypt) (Total cholesterol (TC) [38], triglyceride (TG) [39], and high-density lipoprotein cholesterol (HDL) [40]). Whereas, the low-density lipoprotein cholesterol (LDL) content was computed according to the formula: LDL = (Total cholesterol/1.19 + Triglycerides/1.9 − HDL/1.1–38). Very low-density lipoprotein cholesterol (VLDL) was measured using the turbidimetric method [41]. Serum creatinine [42] and uric acid [43] were measured using an automatic biochemical analyzer (Robotnik Prietest ECO Ambernath (W), Thane, India). Serum total antioxidant capacity (TAC) was determined according to Janaszewska and Bartosz [44] and malondialdehyde (MDA) concentration was measured according to McDonald and Hultin [45].

### 2.5. Hepatic Antioxidant Markers and Heat Shock Protein 70

Livers were collected immediately after slaughter. A portion of each liver was snap-frozen in liquid nitrogen and stored at −80 °C for further analysis. Another portion was vacuum-packed and stored at −20 °C for HSP70 determination. The method of Anderson, et al. [46] was used for the determination of HSP70 level in liver tissue using the enzyme-linked immunoassay.

Total mRNA was extracted and purified from liver tissue samples (~20 mg each) using RNX-Plus reagent (Sinaclon Bioscience, Tehran, Iran) to determine the expression of hepatic superoxide dismutase1 (SOD1), catalase (CAT), and glutathione peroxidase (GPX1). The quantity and quality of RNA were assessed with a NanoDrop^®^ ND–1000 spectrophotometer (NanoDrop Technologies; Wilmington, DE, USA). Complementary DNA (cDNA) was synthesized from the extracted RNA using the Prime-Script RT Reagent Kit (TaKaRa Bio, Inc., Shiga, Japan) following the manufacturer’s instructions. Quantitative real-time PCR (qRT-PCR) was employed on a Rotor Gene Q 6000 system (Qi-agen, Germantown, MD, USA) with gene-specific primers (Table 1). The thermal cycling conditions consisted of an initial denaturation at 95 °C for 30 s, followed by 40 cycles of 94 °C for 40 s, annealing at 64 °C for 35 s, and extension at 72 °C for 30 s. Relative mRNA expression of the target genes was normalized to β-actin, which served as the endogenous reference gene. Normalization was performed by calculating the ratio of the target gene Ct values to those of β-actin, thereby correcting for sample-to-sample variation in RNA input and efficiency [47].

### 2.6. Immunological Parameters

Serum interleukin-1β (IL-1β), interleukin-10 (IL-10), and interferon-γ (INF-γ) concentrations were measured using chicken-specific ELISA kits (MBS2024496, MBS2020250, and MBS2020832, respectively; MyBioSource, San Diego, CA, USA). Serum lysozyme activity was determined [48]. Complement 3 protein (C3) level was determined using a commercial ELISA kit (LS-F9287; LifeSpan Biosciences, Inc., Seattle, WA, USA) following the manufacturer’s guidelines.

### 2.7. Economic Efficiency Evaluation

The economic efficiency of the evaluated heat stress mitigation strategies was calculated based on input costs (including feed and variable production costs) and the output revenue (including final broiler BW), as previously described by Al-Khalaifah, et al. [49]. Prices were repressed in US dollars based on current market rates. Total costs included average variable costs (e.g., chick price, veterinary care, vaccination, and other management expenses) in addition to feed costs. Profitability index, economic efficacy, and return on investment were calculated following the earlier established equations [50].

### 2.8. Statistical Analysis

Statistical analyses were carried out using one-way ANOVA with SPSS Statistics software (v20.0; IBM Corp., Armonk, NY, USA). Tukey’s HSD test was applied for multiple mean comparisons. Graphs were prepared with GraphPad Prism (v8.0). Differences were considered statistically significant at *p* < 0.05. Data are presented as mean values ± pooled standard error of the mean (SEM).

## 3. Results

### 3.1. Productive Parameters

The effects of feed withdrawal (EFW) and vitamin C (Vit C) addition on chickens’ performance indicators under heat stress are summarized in Table 2. Birds in the EFW + Vit C group exhibited a significantly higher final body weight (BW). Moreover, body weight gain (BWG) increased by 5–19% relative to the other investigated groups. This enhancement was also reflected in a significant reduction in FCR. Interestingly, vitamin C-supplemented groups showed an elevation in feed consumption relative to the control group. Although the EFW and Vit C groups showed significant improvements in several performance indicators relative to the control group, the effects were less pronounced than in the combined treatment group. These findings evidently revealed the beneficial effect of combining different stress mitigation strategies on broiler production performance.

### 3.2. Metabolic Hormones and Blood Metabolites

The effects of the investigated management and nutritional strategies on metabolic hormones, blood metabolites, and stress markers are presented in Table 3. The previously noted superiority of the EFW + Vit C group was also reflected in increased circulating metabolic hormone levels. Additionally, this group exhibited elevated serum total protein, albumin, and globulin, along with a reduction in cholesterol levels relative to the control group. These improvements in metabolic hormone circulation and blood metabolite profiles may explain the observed enhancement in production performance. Moreover, the reduction in cholesterol, LDL, VLDL, and triglyceride concentrations observed in birds subjected to our mitigation strategies indicates an improved lipid profile. Conversely, HDL levels significantly increased in response to EFW and Vit C supplementation. These outcomes highlight the beneficial influence of the investigated strategies on blood metabolites and overall bird health.

### 3.3. Antioxidant Status

**Figure 2:**
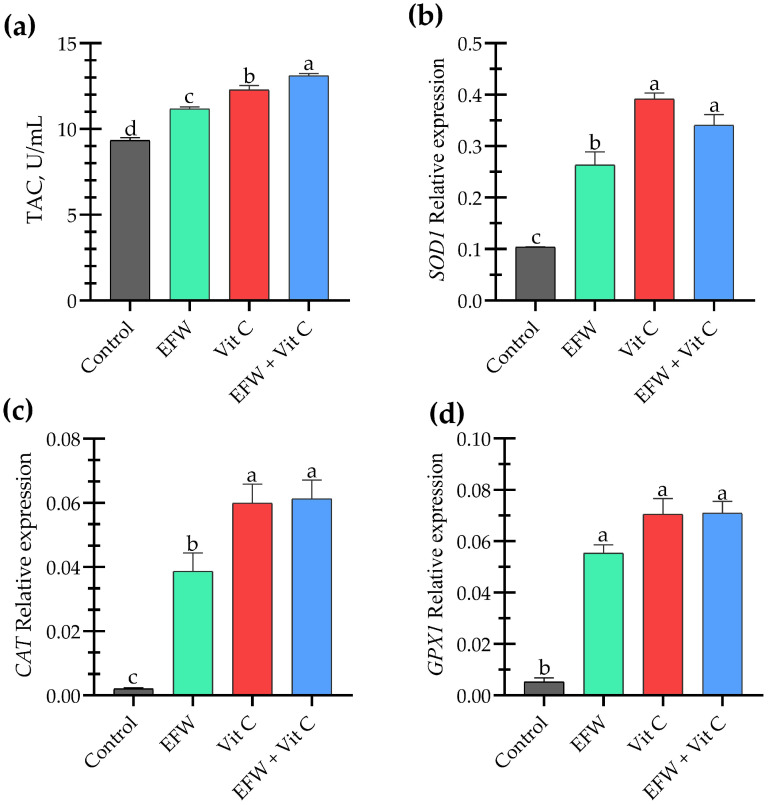
Effects of early feed withdrawal (EFW) and vitamin C (Vit C) supplementation on total antioxidant capacity and relative hepatic gene expression of antioxidant enzymes (**a**) serum total antioxidant capacity (TAC), (**b**) superoxide dismutase 1 (*SOD1*), (**c**) catalase (*CAT*), and (**d**) glutathione peroxidase 1 (*GPX1*) in heat-stressed broiler chickens. Columns with different superscript letters significantly differ (*p* < 0.05).

Endogenous antioxidant enzymes serve as a primary defense system, stabilizing reactive radicals and limiting oxidative harm to cells. The EFW + Vit C group exhibited the highest total antioxidant capacity with a 1.4-fold increase, indicating an overall enhancement in antioxidant activity. The relative gene expression of endogenous hepatic antioxidant enzymes was significantly upregulated in response to EFW and Vit C supplementation compared to the control group (Figure 2). Also, both *SOD1* and *CAT* expression were significantly elevated with vitamin C supplementation and when EFW was compounded with Vit C.

### 3.4. Stress Markers, Heat Shock Protein (HSP70), Cytokines, and Immune Markers

Serum HSP70, cytokines, stress markers, and immune protein markers concentrations are presented in Table 4. During stress, various physiological alterations occur as natural adaptive responses. The stress markers, H/L ratio, and MDA, were significantly reduced in response to EFW and vitamin C supplementation. Meanwhile, HSP70 and anti-inflammatory cytokines are secreted to safeguard cells from oxidative damage and regulate immune function. The HSP70 level was 1.6-fold higher in the vitamin C-supplemented group relative to the control. The experimental groups showed a significant elevation in circulating IL-10 and IL-1β levels relative to the control group, with the EFW + Vit C group showing the highest values. Additionally, IFN-γ level was significantly elevated in the Vit C group, with an even greater increase observed in the EFW + Vit C group. Lysozyme and Complement C3 levels were higher in all experimental groups relative to the control. These findings demonstrate the stress-mitigating and immunomodulation effects of EFW and Vit C supplementation in heat-stressed broilers.

### 3.5. Economic Efficiency

The economic efficiency evaluation revealed a beneficial effect of both EFW and Vit C supplementation on the profitability of heat-stressed broilers. The indicators of the economic evaluation are presented in Table 5. Under our study conditions, the combination of EFW and Vit C resulted in the highest production performance values along with the best feed efficiency. This advantage was reflected in the highest total revenue and net profit per bird. Furthermore, profitability index, rate of return on investment, benefit–cost ratio, and economic efficiency were higher for EFW and EFW + Vit C groups. The calculated relative economic efficiency (REE%) of each treatment group, compared to the control, revealed that the EFW + Vit C group achieved the highest improvement, followed by EFW and Vit C, showing increases of 22, 14, and 10% in economic efficiency, respectively. These findings demonstrate the economic benefits of using EFW combined with Vit C supplementation as promising heat stress mitigation strategies to enhance broiler profitability under hot climatic conditions.

## 4. Discussion

Stress exposure poses a significant threat to modern poultry production, causing detrimental alterations in metabolic performance and blood biochemical parameters that directly affect broiler productivity. Under our experimental conditions, the average minimum THI was 29.2 ± 0.03, while the average maximum THI reached 33.3 ± 0.31. These values indicate severe to very severe stress exposure, especially during the hotter periods of the day. Regardless of broiler breed, HS exposure negatively impacts production performance, resulting in reduced feed intake and lower body weight gain [51,52]. Zeferino, et al. [53] identified the reduction in feed intake as the primary factor contributing to production deterioration under HS. Also, Teyssier, et al. [54] stated that the reduction in feed intake during heat exposure is a direct cause of the changes in broiler performance. In contrast, other investigations reported that the adverse effects of chronic HS are mediated through direct physiological mechanisms rather than solely due to reduced feed intake [55,56]. In the early 1990s, McKee and Harrison [57] declared that vitamin C supplementation improves the growth performance of broilers subjected to various stressors, including HS. Recently, a significant enhancement in BWG and FI was reported in broilers reared under hot conditions and supplemented with 200 mg/kg vitamin C [58]. Additionally, our previous investigation indicated that feed withdrawal during the first week of chicks’ life significantly increased BWG, FI, and feed efficiency [26]. Under the present experiment conditions, the combined application of early feed withdrawal (EFW) and vitamin C supplementation significantly improved broiler performance, resulting in increased FI, higher BWG, and improved FCR. Vitamin C supplementation can enhance body weight gain as well as feed intake in broiler chickens, particularly under heat stress, with the optimal dosage reported to be around 200–300 mg/kg [16,17,59,60]. For example, supplementation with 200 mg/kg vitamin C in drinking water increased feed intake during the first week of heat exposure [60]. Similarly, a dose of 200–500 mg/kg significantly improved BWG and feed intake in broilers subjected to stress [61,62,63,64]. Moreover, several investigations have demonstrated the favorable result of vitamin C supplementation on intestinal morphology, which directly influences nutrient absorption in addition to its role in protein and carbohydrate metabolism [65,66]. Meanwhile, EFW enhances production performance under HS by promoting metabolic adaptation [26]. Combining different stress mitigation strategies led to the greatest improvements in both performance and physiological resilience of heat-stressed broilers.

Thyroid hormones play a crucial role in regulating metabolism. Under HS, a significant metabolic alteration commonly reported is the reduction in circulating thyroid hormone levels, which directly affects metabolism and productivity [26,67,68]. In the present study, the applied stress alleviation strategies elevated both T_3_ and T_4_ levels, particularly in the EFW + Vit C group. This increase in thyroid hormone levels may partially explain the observed improvement in production performance of heat-stressed broilers. Moreover, metabolism involves the absorption and circulation of feed metabolites. Under stress, a disturbance in feed digestion and absorption, as well as nutrient metabolism, was observed. A reduction in blood total protein with an elevation in cholesterol in heat-stressed broilers was reported. The deleterious impact of HS on gut integrity and metabolic hormone circulation directly affects nutrient absorption and overall metabolism [55]. Egbuniwe, et al. [69] indicated that vitamin C added to broilers reared under high environmental temperature increased hemoglobin concentration as well as other hematological parameters. A possible justification of this observation is the ability of vitamin C to enhance iron absorption by reducing Fe^3+^ to Fe^2+^, thereby increasing its bioavailability under heat stress [21]. Moreover, blood metabolic constituents undergo various alterations during HS exposure, reflecting its detrimental effects on physiological and metabolic responses. The changes in lipid metabolism during heat stress were previously investigated. Heat stress is reported to increase the blood total cholesterol level. However, several studies have found that vitamin C supplementation in HS broilers reduces blood lipid constituents (i.e., total cholesterol, triglycerides, and LDL) [70,71,72].

Stress exposure disrupts redox balance by promoting excessive ROS production, which reduces total antioxidant capacity (TAC), impairs antioxidant enzyme activity, and increases lipid peroxidation (MDA). Heat-stressed broilers exhibited a decrease in serum catalase and glutathione peroxidase activity [73]. The investigated strategies individually had a positive impact on redox status. Early feed withdrawal (EFW) enhanced TAC and upregulated the gene expression of hepatic antioxidant enzymes, including *SOD1*, *CAT*, and *GXP1*. Meanwhile, the potent antioxidant activity of vitamin C directly improved redox status, resulting in higher TAC and upregulation of hepatic antioxidant enzyme expression, as previously demonstrated by [22,66]. Notably, the combination of EFW and vitamin C achieved the highest TAC levels, indicating a synergistic effect in mitigating oxidative stress. Moreover, the H/L ratio was reduced in response to EFW and Vit C supplementation, which indicates a stress-relief effect. These findings explain the effective role of EFW and Vit C supplementation in restoring redox homeostasis under HS conditions, which directly affects other physiological responses.

Under unfavorable environmental conditions, Heat shock proteins (HSPs), a group of polypeptides, are secreted to protect proteins from denaturation and maintain cellular homeostasis [74,75]. In response to HS, the expression of HSPs is reported to fluctuate according to different factors such as age, sex, genetics, nutrition, and management [76]. Other studies reported downregulation in blood HSP70 gene expression in response to vitamin C supplementation [70]. The HSPs expression depends on the period of stress exposure and the specific body tissue affected [76]. Under the study conditions, birds were subjected to chronic heat stress, which resulted in a significant elevation in hepatic HSP70 levels in the treated groups relative to the control. Furthermore, the vitamin C-supplemented and EFW+ Vit C groups showed a higher level of HSP70 compared to the EFW group. Hosseindoust, et al. [77] reported that the upregulation of HSP70 mRNA expression reflects the ability of broilers to cope with chronic heat stress. These outcomes indicate that the investigated strategies enhanced broilers’ thermotolerance to varying degrees, depending on their respective modes of action.

Heat stress generally leads to immunosuppression, compromising the bird’s ability to exert an active immune response. Innate immunity is the first barrier of birds’ immune defense, providing a quick, non-specific reaction that is mediated by various components, including complement proteins and cytokines. These molecules are essential mediators of innate immunity, facilitating pathogen recognition and elimination [78,79]. Under oxidative stress induced by heat exposure, uncontrolled inflammation is often triggered, leading to immune suppression [80,81]. Our findings revealed a significant increase in both pro-inflammatory (IL-1β) and anti-inflammatory (IL-10) cytokines. The IL-10 was reported to be significantly up-regulated in the ileum of heat-stressed broilers supplemented with 0.02% vitamin C, while no effect was observed on IFN levels [82]. Additionally, the complement protein C3 and lysozyme levels were elevated in response to the applied stress mitigation strategies. Complement protein C3 is an essential part of the broiler chicken’s immune system, serving an important function in supporting both innate and acquired immune responses [83]. Whereas lysozyme supplementation was reported to enhance immunity, growth performance, and overall antioxidant status in broiler chickens [84]. These molecules are essential for pathogen recognition and elimination, suggesting that early feed withdrawal and vitamin C supplementation, individually or in combination, enhance the innate immune function in heat-stressed broilers [85].

The mechanisms underlying the improvement of physiological and production responses to the studied mitigation strategies are complex. The beneficial effects of early feed withdrawal (EFW) may be partially attributed to early physiological adaptation, which stimulates hepatic HSP70 secretion. Meanwhile, the potent antioxidant properties of vitamin C promote the upregulation of antioxidant enzyme expression and enhance metabolic hormone levels. The enhanced productivity and physiological responses observed with EFW combined with Vit C supplementation are reflected in improved economic efficiency and increased profitability in heat-stressed broiler chicken production.

## 5. Conclusions

This study demonstrated the potential benefits of combining different stress mitigation strategies to enhance broilers’ performance and physiological resilience under heat stress. Early feed withdrawal, when combined with vitamin C supplementation, enhanced broiler production performance through different mechanisms. Vitamin C acted as a potent antioxidant, while early feed withdrawal triggered metabolic adaptations that improved stress tolerance. Therefore, the combination of early feed withdrawal and vitamin C supplementation can be considered an effective strategy to mitigate heat stress and improve broiler productivity and profitability under hot environments. Future research should integrate mitigation strategies that balance environmental sustainability with economic feasibility.

## Figures and Tables

**Table 1:** The primers used for quantitative real-time PCR analysis of chicken endogenous antioxidant enzymes mRNA.

Target Gene		Primers (5′–3′)	PCR Product, bp	Accession No.
*SOD1*	F	CACTGCATCATTGGCCGTACCA	224	NM_205064.1
	R	GCTTGCACACGGAAGAGCAAGT		
*CAT*	F	TGGCGGTAGGAGTCTGGTCT	112	NM_001031215.1
	R	GTCCCGTCCGTCAGCCATTT		
*GPX1*	F	GCTGTTCGCCTTCCTGAGAG	118	NM_001277853.1
	R	GTTCCAGGAGACGTCGTTGC		
*β-actin*	F	AGCGAACGCCCCCAAAGTTCT	139	NM_205518.1
	R	AGCTGGGCTGTTGCCTTCACA		

bp = base pair; *SOD1* = superoxide dismutase 1; *CAT* = catalase; *GPX1* = glutathione peroxidase 1.

**Table 2:** Effects of early feed withdrawal (EFW) and vitamin C (Vit C) supplementation on production performance parameters in heat-stressed broiler chickens.

Parameters	Control	EFW	Vit C	EFW + Vit C	SEM	*p*-Value
Initial BW, g	42.67	42.67	42.67	42.83	0.16	0.982
Final BW, g	1917 ^c^	2091 ^b^	2164 ^b^	2281 ^a^	29.04	<0.0001
BWG, g	1875 ^c^	2048 ^b^	2121 ^b^	2238 ^a^	29.05	<0.0001
Feed intake, g	3172 ^b^	3300 ^ab^	3404 ^a^	3363 ^a^	24.66	0.001
FCR	1.69 ^a^	1.61 ^b^	1.60 ^b^	1.50 ^c^	0.02	<0.0001

Means within the same row that differ significantly are indicated by distinct superscript letters (*p* < 0.05). BW: body weight; BWG: body weight gain; FCR: feed conversion ratio; SEM: standard error of the mean.

**Table 3:** Effects of early feed withdrawal (EFW) and vitamin C (Vit C) supplementation on metabolic hormones, blood metabolites, and heat stress markers in heat-stressed broiler chickens.

Parameters	Control	EFW	Vit C	EFW + Vit C	SEM	*p*-Value
T_3_, ng/dL	3.61 ^b^	3.32 ^c^	4.04 ^a^	4.15 ^a^	0.07	<0.0001
T_4_, ng/dL	21.59 ^c^	23.59 ^b^	23.71 ^b^	24.65 ^a^	0.25	<0.0001
Hemoglobin, g/dL	9.11 ^c^	9.74 ^b^	10.61 ^a^	11.09 ^a^	0.17	<0.0001
Total protein, g/dL	5.30 ^c^	6.13 ^b^	6.38 ^ab^	6.73 ^a^	0.12	<0.0001
Ablumin, g/dL	2.71 ^b^	2.80 ^ab^	3.36 ^a^	3.35 ^a^	0.09	0.0043
Globulin, g/dL	2.59 ^b^	3.32 ^a^	3.02 ^a^	3.37 ^a^	0.08	<0.0001
T-Chol, mmol/L	3.56 ^a^	3.32 ^b^	3.23 ^bc^	3.20 ^c^	0.03	<0.0001
HDL, mmol/L	2.03 ^b^	2.22 ^a^	2.28 ^a^	2.29 ^a^	0.02	<0.0001
LDL, mmol/L	1.28 ^a^	0.88 ^b^	0.73 ^c^	0.69 ^c^	0.05	<0.0001
VLDL, mmol/L	0.25 ^a^	0.23 ^b^	0.22 ^b^	0.22 ^b^	0.003	<0.0001
Triglycerides, mmol/L	1.32 ^a^	1.23 ^b^	1.22 ^b^	1.18 ^b^	0.01	<0.0001
Uric Acid, mg/100 mL	3.25	2.83	2.90	2.76	0.09	0.1943
Creatinine, mg/dL	0.280 ^a^	0.260 ^ab^	0.255 ^ab^	0.238 ^b^	0.005	0.045

Means within the same row that differ significantly are indicated by distinct superscript letters (*p* < 0.05). T_3_: triiodothyronine; T_4_: thyroxin; T-Chol: total cholesterol; HDL: high density lipoprotein; LDL: low density lipoprotein; VLDL: very low density lipoprotein; SEM: standard error of the mean.

**Table 4:** Effects of early feed withdrawal (EFW) and vitamin C (Vit C) supplementation on stress markers, Heat shock protein 70, anti-inflammatory, pro-inflammatory cytokines, and immune factors in heat-stressed broiler chickens.

Parameters	Control	EFW	Vit C	EFW + Vit C	SEM	*p*-Value
MDA, nmol/mL	5.61 ^a^	2.83 ^b^	2.34 ^c^	2.26 ^c^	0.29	<0.0001
H/L ratio	0.61 ^a^	0.54 ^b^	0.52 ^b^	0.51 ^b^	0.01	<0.0001
HSP70, ng/mg	3.81 ^c^	5.11 ^b^	6.21 ^a^	6.09 ^a^	0.21	<0.0001
IFN-γ, pg/mL	6.70 ^c^	7.40 ^c^	9.15 ^b^	10.53 ^a^	0.34	<0.0001
IL-10, µg/mL	1.60 ^c^	2.93 ^b^	3.38 ^b^	3.97 ^a^	0.19	<0.0001
IL-1β, µg/mL	143.0 ^c^	160.2 ^b^	169.8 ^a^	170.8 ^a^	2.50	<0.0001
Lysozyme, µg/mL	131.0 ^b^	166.3 ^a^	179.0 ^a^	184.2 ^a^	4.82	<0.0001
Complement C3, g/L	1.21 ^b^	1.44 ^a^	1.51 ^a^	1.50 ^a^	0.03	<0.0001

Means within the same row that differ significantly are indicated by distinct superscript letters (*p* < 0.05). MDA: malondialdehyde; H/L ratio: heterophils to lymphocytes ratio; HSP70: heat shock protein 70; IFN-γ: Interferon-Gamma; IL-10: Interleukin 10; IL-1β: Interleukin 1 Beta; SEM: standard error of the mean.

**Table 5:** Effects of early feed withdrawal (EFW) and vitamin C (Vit C) supplementation on economic efficiency indicators in heat-stressed broiler chickens.

Indicators	Control	EFW	Vit C	EFW + Vit C	SEM	*p*-Value
Feed cost/kg diet *	0.45	0.45	0.50	0.500	---	---
Feed cost/kg BWG	0.761 ^b^	0.726 ^c^	0.802 ^a^	0.752 ^bc^	0.01	<0.0001
Total feed cost/bird	1.427 ^b^	1.485 ^b^	1.702 ^a^	1.681 ^a^	0.03	<0.0001
Total cost/bird	1.687 ^b^	1.745 ^b^	1.962 ^a^	1.941 ^a^	0.03	<0.0001
Total revenue/bird **	3.835 ^c^	4.182 ^b^	4.328 ^b^	4.562 ^a^	0.06	<0.0001
Net profit	2.147 ^c^	2.437 ^b^	2.366 ^b^	2.620 ^a^	0.04	<0.0001
Profitability index	0.560 ^bc^	0.582 ^a^	0.547 ^c^	0.574 ^ab^	0.004	<0.0001
Economic efficiency%	150.6 ^bc^	164.3 ^a^	139.1 ^c^	155.9 ^ab^	2.39	<0.0001
REE%	100 ^c^	114 ^b^	110 ^b^	122 ^a^	3.22	<0.0001
RRI%	127.37 ^bc^	139.75 ^a^	120.63 ^c^	135.00 ^ab^	1.92	<0.0001
BCR	2.274 ^bc^	2.397 ^a^	2.206 ^c^	2.350 ^ab^	0.019	<0.0001
Profit margin%	56.00 ^bc^	58.24 ^a^	54.66 ^c^	57.43 ^ab^	0.35	<0.0001

Means within the same row that differ significantly are indicated by distinct superscript letters (*p* < 0.05). Variable costs of one bird included; labor, veterinarian care, vaccination, and other management costs were estimated to be $0.26 US; selling costs of kg live body weight = $2 US. * Feed cost including vitamin C. ** Total revenue = final BW × $2 US. Net profit = total revenue − total expenses. Profitability index = net profit/total revenue. RRI% (Rate of Return on Investment%) = net profit/total cost × 100. Economic efficiency% = net profit/total feed cost × 100. REE% (relative economic efficiency%) = net profit per treatment group/net profit per control group × 100; BCR (Benefit–cost ratio) = total revenue/total cost; Profit margin% = net profit/total revenue × 100. BWG: body weight gain; SEM: standard error of the mean.

## Data Availability

The original contributions presented in this study are included in the article. Further inquiries can be directed to the corresponding authors.

## Abbreviations

The following abbreviations are used in this manuscript:

Alb	Albumin
BW	Body weight
BWG	Body weight gain
CAT	Catalase
Chol	Cholesterol
EFW	Early feed withdrawal for 24 h
FCR	Feed conversion ratio
FI	Feed intake
Glob	Globulin
GXP	Glutathione peroxidase
H/L ratio	Heterophils to lymphocytes ratio
Hb	Hemoglobin
HSP70	Heat shock protein 70
IFN-γ	Interferon-gamma
IL-10	Interleukin 10
IL-1β	Interleukin 1 Beta
SOD	Superoxide dismutase
T_3_	Triiodothyronine
T_4_	Thyroxin
TAC	Total antioxidant capacity
TP	Total protein
Vit C	Vitamin C
